# An unusual case of intra orbital foreign body; diagnosis, management, and outcome: a case report

**DOI:** 10.1186/s12893-019-0536-2

**Published:** 2019-07-04

**Authors:** Farhad Mirzaei, Firooz Salehpour, Ghaffar Shokuhi, Touraj Asvadi Kermani, Sana Salehi, Sina Parsay

**Affiliations:** 1 Department of Neurosurgery, Faculty of Medicine, Tabriz University of Medical Sciences, Tabriz, Iran; 2 Trauma Surgery, Department of General and Vascular Surgery, Faculty of Medicine, Tabriz University of Medical Science, Tabriz, Iran; 3 School of Medicine, Mercer University, Savannah, GA USA; 4 Student Research Committee, Tabriz University of Medical Science, Tabriz, Iran

**Keywords:** Head trauma, Intraorbital foreign body, Craniotomy, CT

## Abstract

**Background:**

An orbitocranial injury with a penetrating Intraorbital Foreign Body (IOFB) is listed as a rare cause of penetrating trauma. Since this type of trauma is considered a surgical emergency, taking a thorough history along with careful examination to find out the mechanism and cause of the trauma is crucial towards correct diagnosis and management of the disease.

**Case presentation:**

A 35-year-old male patient was presented to the ER with an occupational craniofacial injury because of an IOFB. The patient underwent an extra-dural orbitocranial craniotomy procedure to remove the foreign body. Interestingly, a plastic foreign body (a piece of a plastic pipe) was removed from the orbital cavity, which was suspected to be a fractured orbital bone, at first place.

**Conclusion:**

In this study, we demonstrated that plastics could mimic bone structure in a Computerized Tomography (CT) scan leading to possible initial misdiagnosis. Hence high clinical suspicion is necessary for the correct diagnosis of such cases. However, despite the prompt intervention, our patient ended up with permanent vision loss in his injured eye.

## Background

Orbitocranial injury with a penetrating Intraorbital Foreign Body (IOFB) is considered a rare cause of penetrating trauma. It commonly occurs among young people and is usually associated with high-velocity craniofacial trauma forces like occupational injuries, gunshot wounds or even simpler injuries such as daily household chores [1, 2].

Since a craniofacial trauma, especially an IOFB to the optic nerve, is considered a surgical emergency, immediate diagnosis and management plan is mandatory.

We present a rare case of occupational head trauma with a large IOFB which led to a marked visual impairment and optic nerve dysfunction, despite the small size of the laceration. What added to the value of this case was an unusual appearance in CT scan that could have misguided the physicians, initially.

## Case presentation

The patient was a 35-year-old male presented to the ER with right craniofacial trauma due to an occupational injury caused by falling of a plastic pipe on his head. He was awake and aware at the time of arrival. The initial assessment revealed a GCS level of 14. Signs of traumatic injuries in the right side of his face included swollen eyelids due to trauma to the soft tissue of the right periorbital and frontal area along with right superior eyelid laceration, redness, and tenderness. The visual acuity in his right eye was almost no light perception. Right optic neuropathy was evident with a relative afferent pupillary defect (RAPD) of 4+. The eye movements of the right eye were restricted in all directions, but the other side was normal. Other examinations revealed no further abnormal findings.

**Fig. 1:**
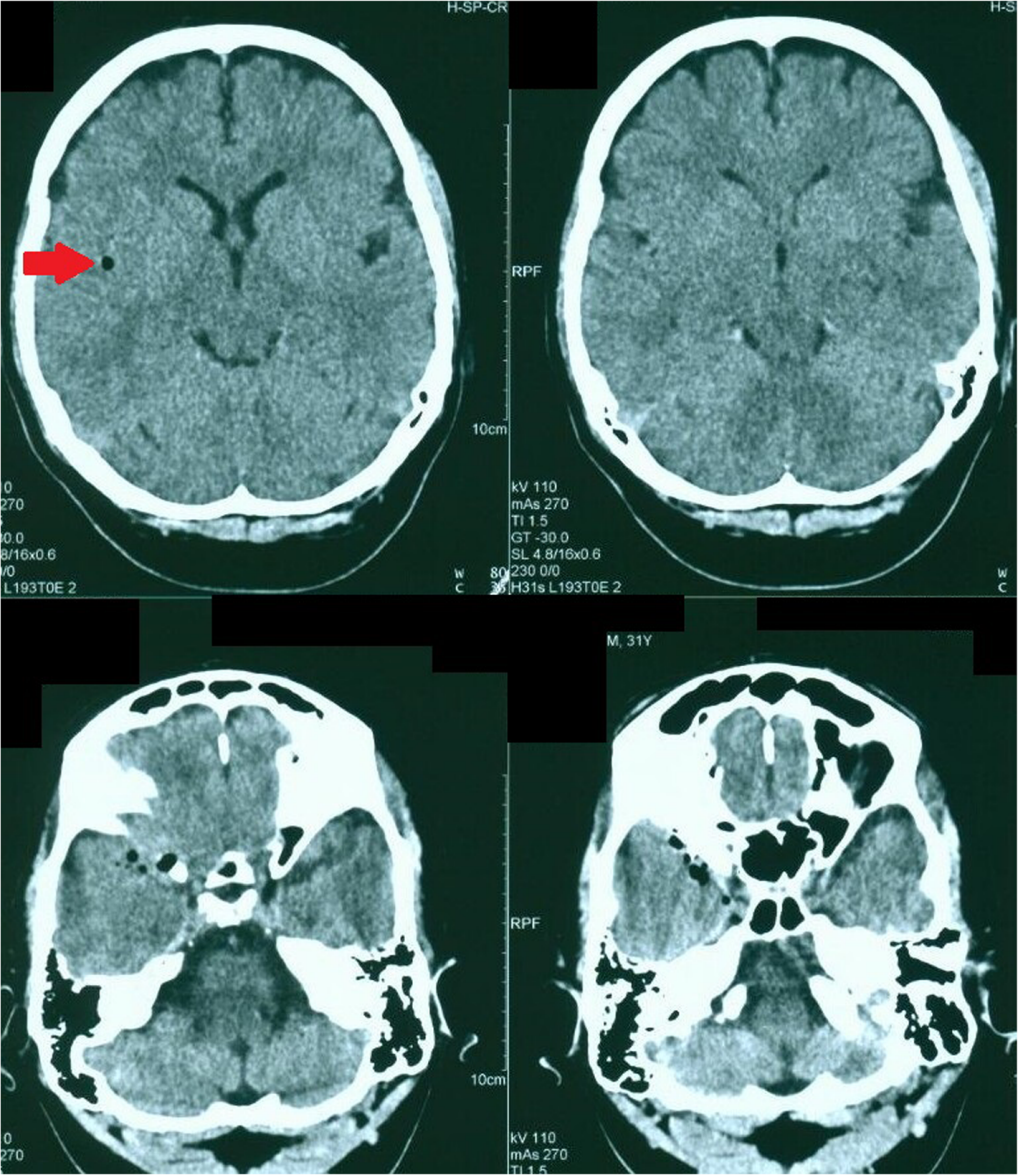
Brain CT, Parenchyma Window, Axial Images. arrow demonstrates a focus of pneumocephalus in right sylvian area

**Fig. 2:**
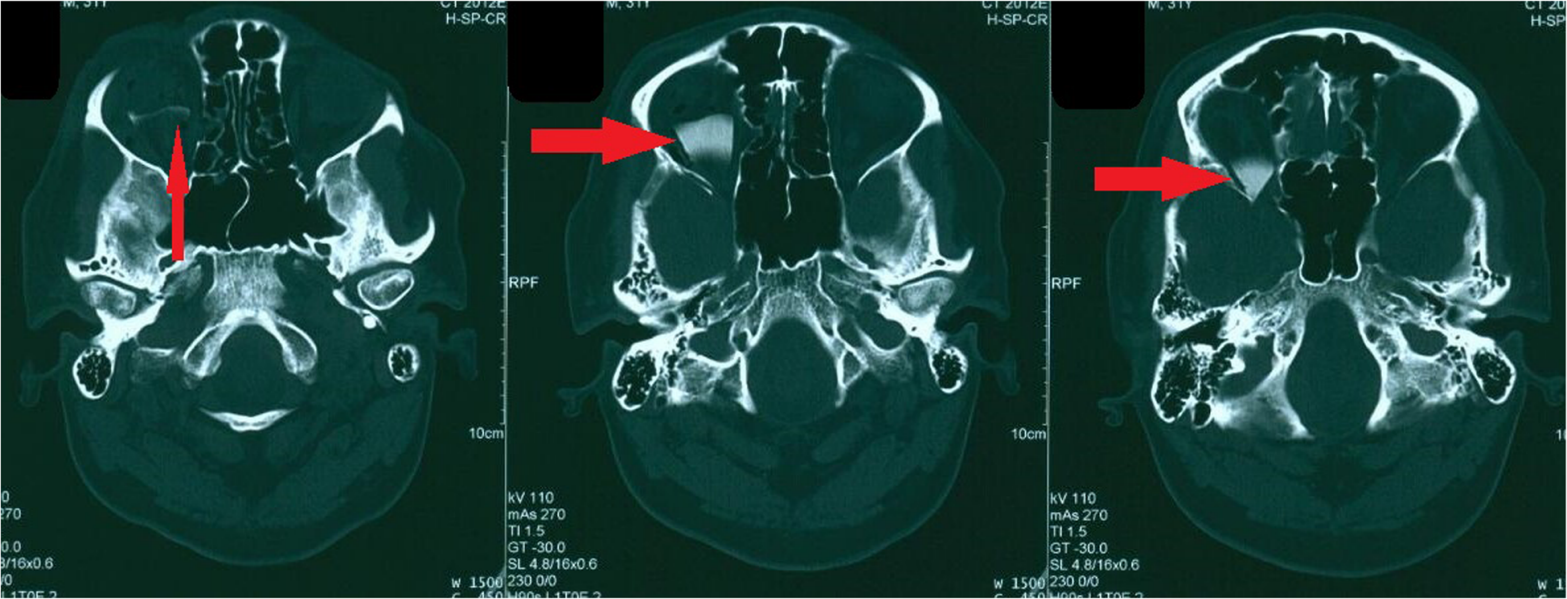
Brain CT, Bone Window, Level of Orbital Cavity, Axial Images. Image demonstrates a hyperdens area posterior to the right globe mimicking a boney structure

**Fig. 3:**
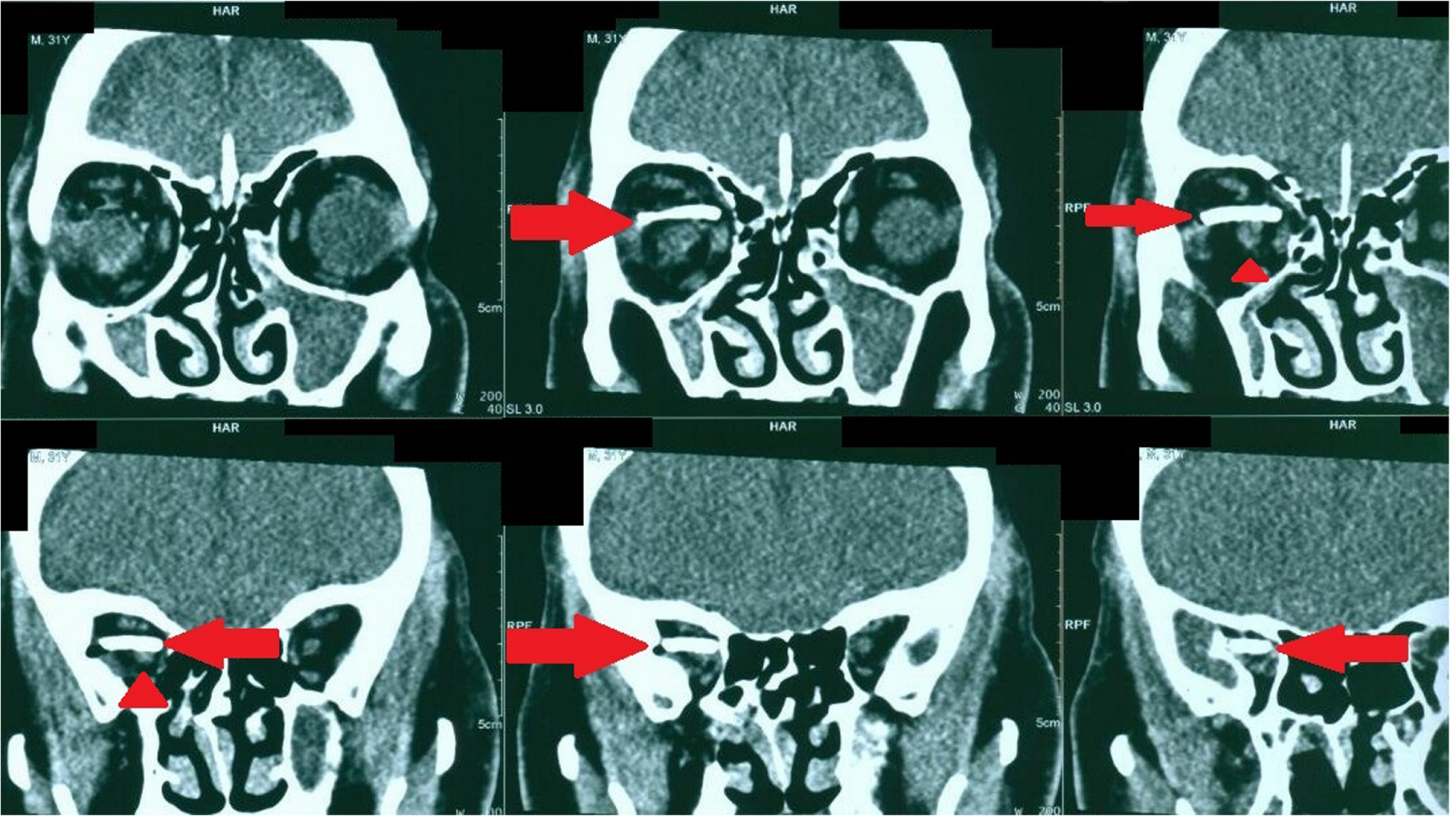
Coronal Reconstructed Images from Region of Orbital Cavity. (foreign body demonstrated by arrows and arrow head shows optic nerve)

**Fig. 4:**
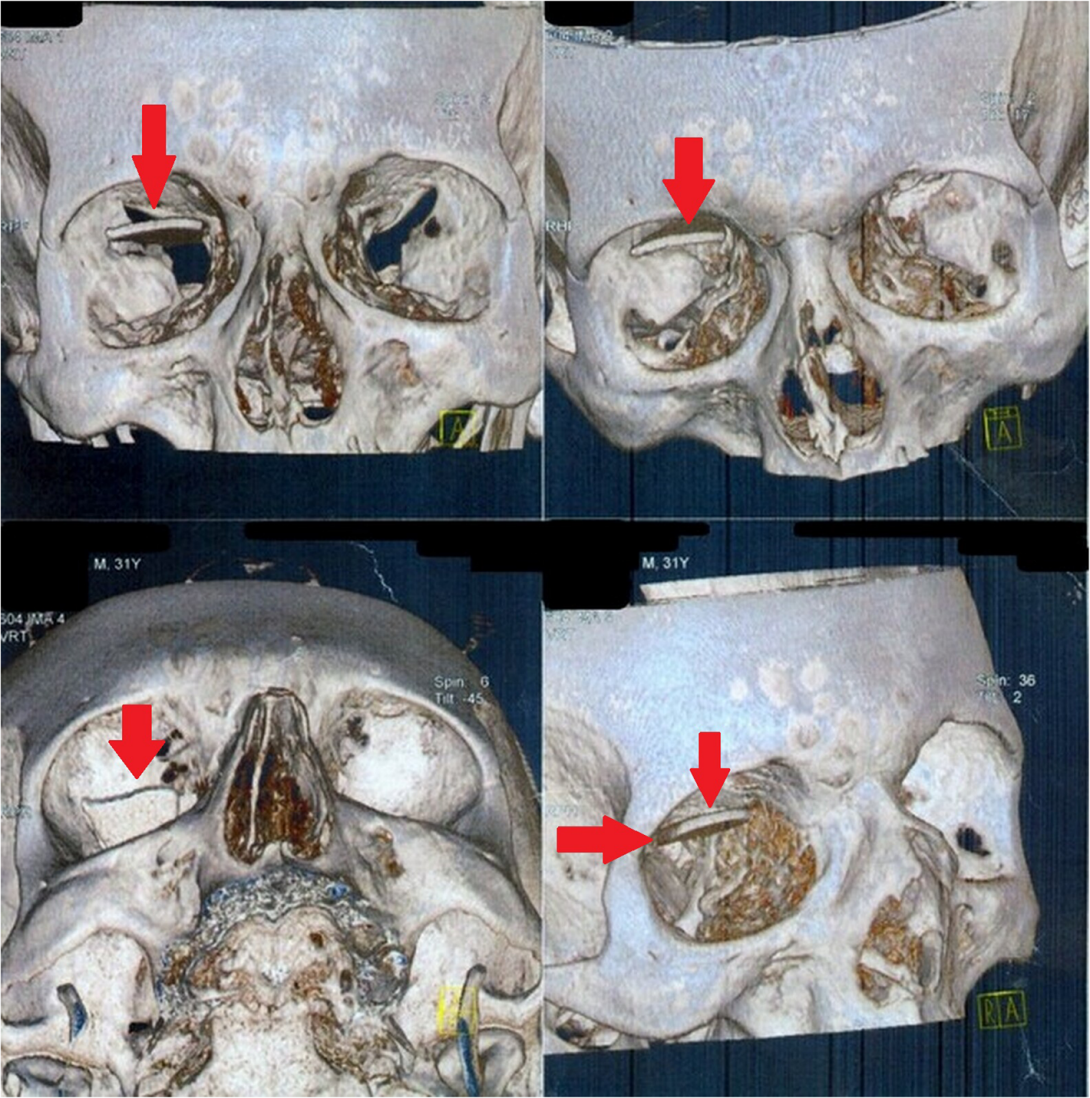
3D-Reconstructed Head CT (the object is seen in right orbital fossa)

**Fig. 5:**
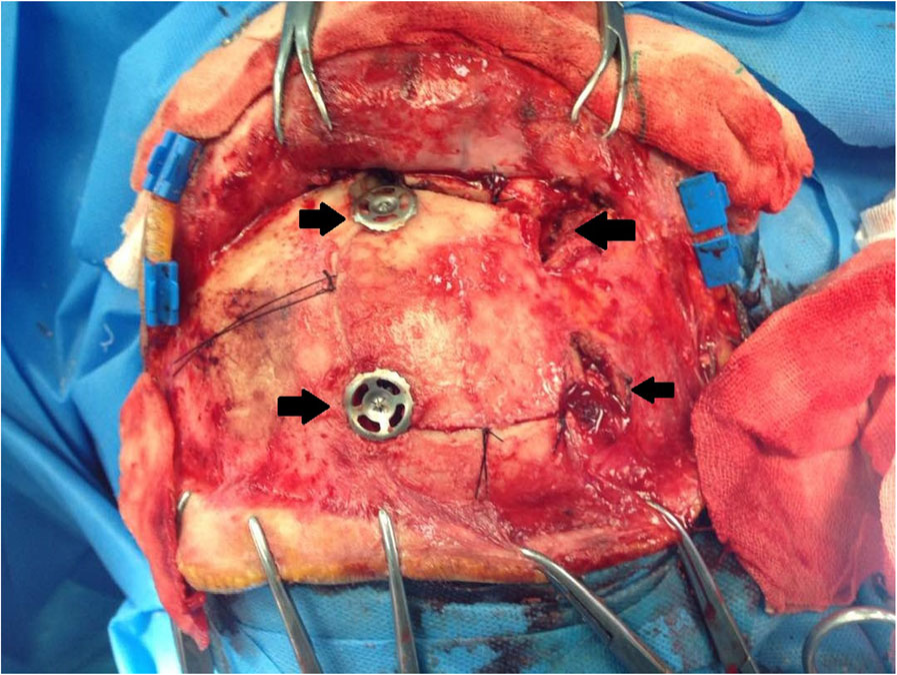
Initiating Frontal Craniotomy by trephination of 4 burr holes (arrows demonstrate burr holes)

**Fig. 6:**
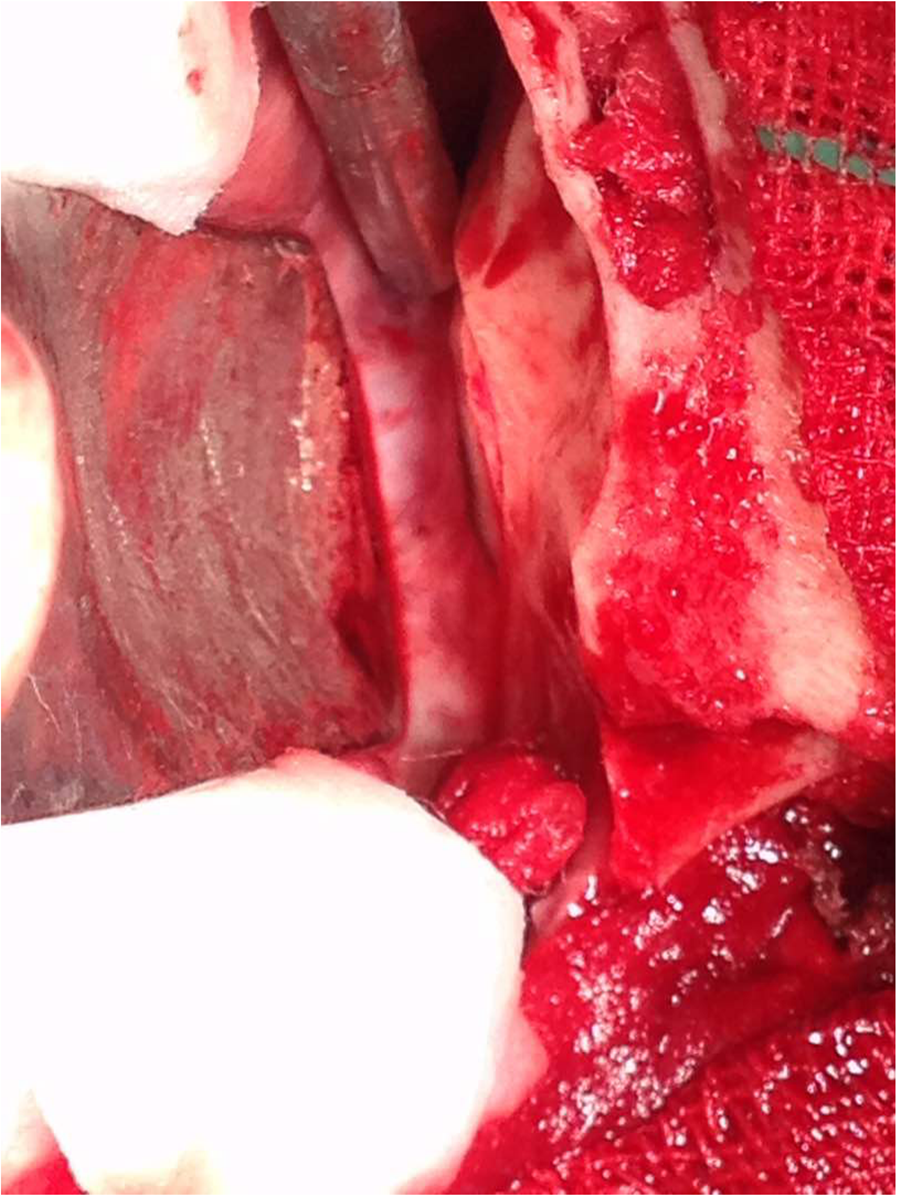
Extending the surgical field into the orbital cavity and dissecting the area

**Fig. 7:**
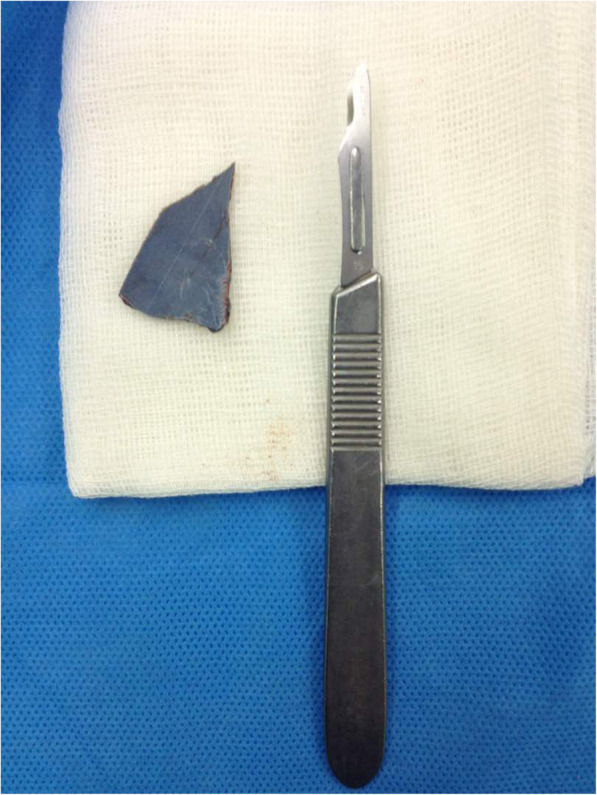
Plastic foreign body excised from the orbital cavity

**Fig. 8:**
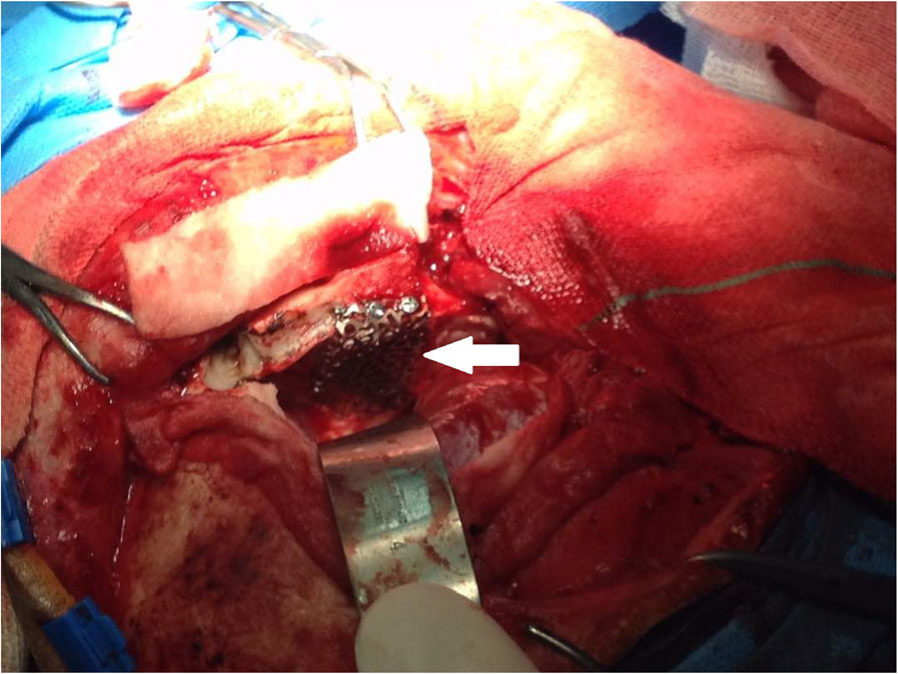
Performing cranioplasty with titanium mesh

CT-Scan depicted a small focus at the level of the right Sylvian fissure in favor of a pneumocephalus and a hyperdense structure in the right orbital cavity, posterior to the globe, suggestive of a bony material (Figs. 1, 2, 3 and 4). As we were not sure about the origin and material of hyperdense structure demonstrated on CT-Scan, use of MRI was waived in order to prevent subsequent complications in case of metal object foreign body. However, initial assessments of patient’s images amplified the suspicion of skull base fracture, regarding the foci of pneumocephalus, especially at the region of the right superior orbital wall. Based on these findings, the patient was admitted to the trauma section of the neurosurgery ward and received initial necessary supportive care. Antibiotic therapy was commenced for surgery preparation and preventing probable meningitis, with Cefepime and Vancomycin. A consult with ophthalmologists was performed, and regarding their evaluation, both ophthalmologists and neurosurgeons were agreeing with choosing a craniotomy approach. The procedure was performed by using the method of “extra-dural orbitocranial approach to the anterior cranial fossa” craniotomy. After a right brow skin incision with a soft tissue dissection with four burr holes trephination, a right frontal craniotomy was performed. Then, the roof of the orbital cavity was explored. No signs of fracture were detected in the superior orbital wall. To asses the orbital cavity, a supraorbital craniotomy was carried out, and the surgical field was extended to the right orbital cavity by removing the superior orbital wall, with a diameter of 1.5 × 2.5 cm. An exploration and dissection throughout the intraorbital muscles and structures were accomplished (Figs. 5, 6). An intraorbital foreign body was successfully (a piece of plastic pipe) removed from the orbital cavity (Fig. 7). Cranioplasty was carried out by titanium mesh and bone wax with wound closure as well (Fig. 8). The patient was transferred to the neurosurgery ward to receive his post-op cares and subsequent follow-ups.

On microbiological culture of resected specimen colonies of *Staphylococcus epidermidis* were isolated which was sensitive to given antibiotics.

The patient was febrile, thus antibiotic therapy was continued with Cefepime and Vancomycin for 7 days based on the consult performed by an infectious disease specialist. In the patient’s initial evaluation after the operation, the overall condition was good, and his GCS level was 15. Eye-movements were normal on both sides. Regarding the patient’s visual loss and its effect on his quality of life in order to treat the consequences of traumatic optic neuropathy (TON) both ophthalmologists and neurosurgeons were agreeing with the administration of intravenous methylprednisolone (IVMP) with close monitoring of the patient. The patient received 250 mg methylprednisolone intravenously every 6 h for 3 days. Despite the foreign body removal and administration of high dose IVMP, patient’s visual acuity in his right eye was merely confined to light perception and did not improve, subsequently.

We presume that the patient’s vision loss occurred due to direct and high impact trauma to the optic nerve based on the location of the foreign body, which was embedded in the posterior part of the globe, just beside the optic nerve (arrow heads on Figure-3).

The patient was discharged from hospital 2 weeks after admission with a good general condition.

## Discussion and conclusion

The clinical manifestation, management, and outcomes of orbital foreign bodies vary based on the material of the foreign body [3].

Based on their composition, these objects can be categorized as three distinct types; 1- metal objects such as aluminum, steel, and iron; 2- inorganic-nonmetallic objects such as plastic, glass, rock, etc.; and 3) organic objects such as wood, plants, thorns, and vegetable matter [4].

The common sites for a penetrating foreign body within the orbit are superior (26%), medial (30%), inferior (26%), and lateral wall (4%).

Orbital traumas that are associated with an optic nerve injury, either directly or indirectly, should always be considered as a surgical emergency. In such cases, early removal of the foreign body within 12–24 h after the injury is essential [5]; therefore, high clinical suspicion, careful physical examination, and choosing appropriate imaging modalities are necessary to prevent misdiagnosis.

Our case demonstrates that despite prompt intervention and removal of the foreign body, permanent visual impairment is inevitable, sometimes. The visual impairment might happen because of the high intensity of the trauma and the location of the foreign body.

CT is generally considered as the choice imaging modality for detecting IOFBs. It is considered a safe modality when it comes to metallic objects. On the other hand, excluding the orbitocranial extension, and diagnosing orbital wall fractures could be counted as advantages of the CT scan [6–8].

Some advantages of spiral CT in comparison to conventional scans include: providing high quality coronal, sagittal, and 3-Dimensional (3D) reconstructed images, reducing motion artifact, lower radiation exposure to the lens, and reducing artifact from metallic objects [9].

However, CT scan sometimes could have misleading results due to the different composition of foreign body materials. As we mentioned earlier, we represented a case of plastic IOFB which appeared as a bony object in CT. Therefore, taking a thorough history of the incident and precise physical examination tend to be the substantial prerequisites towards reaching a correct clinical diagnosis.

On the other hand, the surgical approach is selected based on the nature of the foreign body, its location, and its complications. Foreign bodies of the 2/3 anterior of the orbital cavity might be approached extra-cranially. In contrast, objects located in the apical area, require a transcranial approach. Furthermore, Orbital injuries extending beyond the superior orbital fissure need a combined and complicated surgical procedure due to their proximity to the cavernous sinus [1].

Removal of the foreign body through the anterior approach always bears a risk of significant bleeding and mortality caused by the abrupt removal of the tamponing effect applied by the foreign body to the lacerated area. Since the transcranial approach can provide better control to the neurovascular structures, this method is considered as the procedure of choice if there are any findings in favor of major neurovascular injury [8].

In this study, we demonstrated a procedure with an extra-dural orbitocranial approach to the anterior cranial fossa for removing an intraorbital foreign body.

In conclusion, in cases of craniofacial trauma, we suggest thorough history taking, precise physical examination, and a high clinical suspicion for IOFB towards reaching an early diagnosis.

On the other hand, paraclinical studies sometimes may be misleading, and some objects might have various manifestations in imaging studies.

Our patient underwent transcranial removal of the IOFB due to its location. We scheduled a follow-up visit to the clinic 2 weeks after his discharge. His was doing well on the path of recovery, but visual acuity still had remained as light perception.

## Consent

Written informed consent was obtained from the patient for publication of this case report and any accompanying images. A copy of the written consent is available for review by the Editor-in-Chief of this journal.

## Data Availability

All data is contained within the manuscript and its additional files.

## Abbreviations

3D: Three Dimensional
CT: Computerized Tomography
GCS: Glasgow Coma Scale
IOFB: Intraorbital Foreign Body
IVMP: Intra Venous Methylprednisolone
RAPD: Relative Afferent Pupillary Defect
TON: Traumatic Optic Neuropathy

## References

[1] Turliuc DM, Costan V, Cucu A, Costea CF (2015). Intraorbital foreign body. Med Surg J.

[2] Türkçuoğlu P, Aydoğan S (2006). Intracranial foreign body in a globe-perforating injury. Can J Ophthalmol.

[3] Fulcher TP, McNab AA, Sullivan TJ (2002). Clinical features and management of intraorbital foreign bodies. Ophthalmology.

[4] Moretti A, Laus M, Crescenzi D, Croce A (2012). Peri-orbital foreign body: a case report. J Med Case Rep.

[5] Hamilton A, Meena M, Lawlor M, Kourt G (2014). An unusual case of intraorbital foreign body and its management. Int Ophthalmol.

[6] Rubinstein A, Riddell CE, Kafil-Hussain N, Assaf A (2005). Self-inserted intraorbital foreign bodies. Ophthalmic Plast Reconstr Surg.

[7] Howe L, Jones N (2004). Guidelines for the management of periorbital cellulitis/abscess. Clin Otolaryngol Allied Sci.

[8] Singh A, Sadique SI, Ghosh SN (2018). Intraorbital foreign body with intracranial extension: a case series. International Clinical Neuroscience Journal.

[9] Adesanya O, Dawkins DM (2007). Intraorbital wooden foreign body (IOFB): mimicking air on CT. Emerg Radiol.

